# Resistance to Inhibitors of Cholinesterase 3 (Ric-3) Expression Promotes Selective Protein Associations with the Human α7-Nicotinic Acetylcholine Receptor Interactome

**DOI:** 10.1371/journal.pone.0134409

**Published:** 2015-08-10

**Authors:** Matthew J. Mulcahy, Sydney B. Blattman, Francisco J. Barrantes, Ronald J. Lukas, Edward Hawrot

**Affiliations:** 1 Department of Molecular Pharmacology, Physiology and Biotechnology, Brown University, Providence, Rhode Island, United States of America; 2 Laboratory of Molecular Neurobiology, Institute of Biomedical Research, UCA-CONICET, Buenos Aires, Argentina; 3 Division of Neurobiology, Barrow Neurological Institute, Phoenix, Arizona, United States of America; Chiba University Center for Forensic Mental Health, JAPAN

## Abstract

The α7-nicotinic acetylcholine receptor (α7-nAChR) is a ligand-gated ion channel widely expressed in vertebrates and is associated with numerous physiological functions. As transmembrane ion channels, α7-nAChRs need to be expressed on the surface of the plasma membrane to function. The receptor has been reported to associate with proteins involved with receptor biogenesis, modulation of receptor properties, as well as intracellular signaling cascades and some of these associated proteins may affect surface expression of α7-nAChRs. The putative chaperone resistance to inhibitors of cholinesterase 3 (Ric-3) has been reported to interact with, and enhance the surface expression of, α7-nAChRs. In this study, we identified proteins that associate with α7-nAChRs when Ric-3 is expressed. Using α-bungarotoxin (α-bgtx), we isolated and compared α7-nAChR-associated proteins from two stably transfected, human tumor-derived cell lines: SH-EP1-hα7 expressing human α7-nAChRs and the same cell line further transfected to express Ric-3, SH-EP1-hα7-Ric-3. Mass spectrometric analysis of peptides identified thirty-nine proteins that are associated with α7-nAChRs only when Ric-3 was expressed. Significantly, and consistent with reports of Ric-3 function in the literature, several of the identified proteins are involved in biological processes that may affect nAChR surface expression such as post-translational processing of proteins, protein trafficking, and protein transport. Additionally, proteins affecting the cell cycle, the cytoskeleton, stress responses, as well as cyclic AMP- and inositol triphosphate-dependent signaling cascades were identified. These results illuminate how α-bgtx may be used to isolate and identify α7-nAChRs as well as how the expression of chaperones such as Ric-3 can influence proteins associating with α7-nAChRs. These associating proteins may alter activities of α7-nAChRs to expand their functionally-relevant repertoire as well as to affect biogenesis and membrane trafficking of α7-nAChRs.

## Introduction

The α7-nicotinic acetylcholine receptor (α7-nAChR) is a homopentameric ligand-gated ion channel widely expressed in both neuronal and non-neuronal tissue and is associated with numerous physiological processes such as memory and cognition [1]. Compared to other nAChR subtypes, the α7-nAChR desensitizes more rapidly, is more permeable to Ca^2+^, and is a target for highly selective ligands such as α-Bungarotoxin (α-bgtx), derived from the venom of the snake *Bungarus multicinctus* and methyllycaconitine (MLA), derived from plants of the *Delphinium* genus [2–4]. These highly selective ligands are powerful tools that enable the isolation of α7-nAChRs and associated proteins.

Receptor-protein associations can occur at various stages of a receptor’s life-cycle to facilitate receptor assembly and intracellular trafficking to and from the cell surface membrane, to modulate receptor function, and to play a role in cellular signaling [3, 5]. Proteins and classes of proteins associating with nAChRs have been reported that affect each of these processes, in particular those processes which facilitate receptor assembly and trafficking [3, 6, 7]. Specifically, chaperones and proteins that affect post-translational modifications such as disulfide bond formation, dephosphorylation, palmitoylation, and glycosylation have been associated with nAChR assembly and trafficking [3]. Associating proteins that are involved in the complex process of α7-nAChR surface expression are of particular interest because alterations in nAChR expression can contribute to disease [8–16]. Additionally, one of the limited number of proteins previously reported to associate with α7-nAChRs, is the molecular chaperone resistance to inhibitors of cholinesterase 3 (Ric-3), which has been shown to facilitate nAChR assembly and trafficking [9, 17].

Ric-3 is a chaperone that is predominantly localized to the endoplasmic reticulum (ER) and has been shown to increase functional expression of homomeric α7-nAChRs on the cell surface [8, 9, 18–23]. Ric-3 also has been reported to enhance the expression of α8-, α9-, α3β4-, α3β2-, α4β2-, and α4β4-nAChRs in mammalian cells [24]. The mechanisms by which Ric-3 enhances surface expression of α7-nAChRs are not fully understood. One proposed mechanism is that Ric-3 promotes the assembly of nAChR subunits into complete oligomers to facilitate transportation of α7-nAChRs out of the ER [8, 9, 23, 25]. It has also been suggested that the expression of Ric-3 may be necessary for the recruitment of additional associated proteins to facilitate nAChR surface expression [24].

The SH-EP1-hα7-Ric-3 cell line has been developed as a model for studies of stable surface expression of functional human α7-nAChRs [9]. The parental, human tumor-derived SH-EP1 epithelial cell line expresses little, if any, α7-nAChRs or Ric-3 [26, 27]. Capitalizing on the lack of endogenous expression, the SH-EP1-hα7 cell line was established to stably express human α7-nAChRs [28]. In a second round of transfection, the SH-EP1-hα7-Ric-3 cell line was established to provide stable Ric-3 protein expression and was shown to express a substantially higher level of functional α7-nAChRs on the cell surface [9].

Work by Paulo *et al*. used α-bgtx-affinity purification and mass spectrometry to identify proteins of the murine brain α7-nAChR interactome, i.e., proteins either interacting with the α7-nAChR or associated with the α7-nAChR protein complex [29]. The work described here uses α-bgtx-affinity to purify α7-nAChR protein complexes, reproducibly identify human α7-nAChR peptides, and identifies associated proteins mediated by Ric-3 expression using high-throughput mass spectrometry.

**Fig 1:**
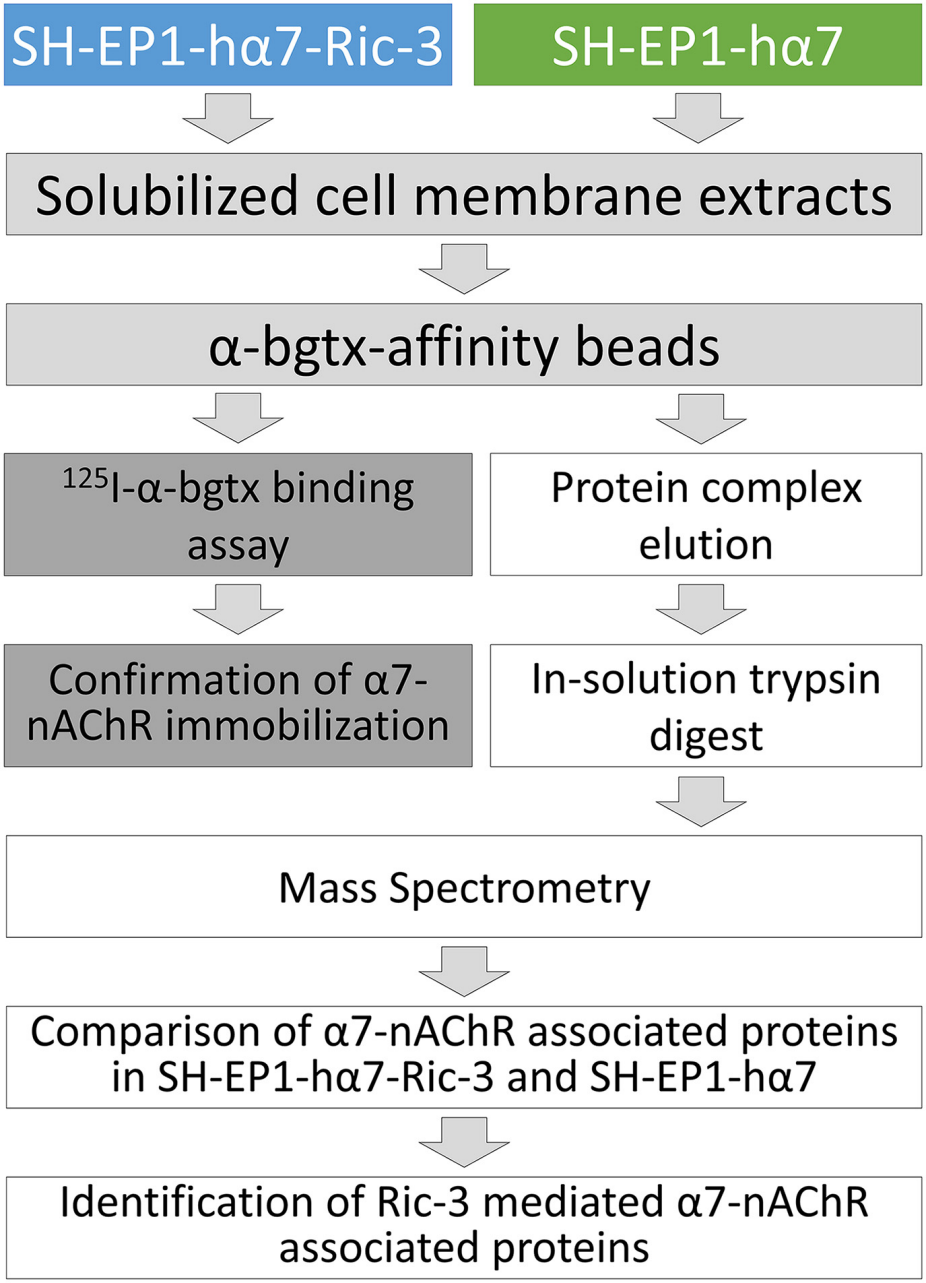
Experimental design. Five biological replicates of both SH-EP1-hα7-Ric-3 cells and SH-EP1-hα7 cells were independently processed and analyzed. Triton X-100 solubilized α7-nAChR protein complexes were isolated from SH-EP1-hα7-Ric-3 and SH-EP1-hα7 extracts using α-bgtx-affinity beads. Binding of α7-nAChRs to affinity beads was confirmed with ^125^I-α-bgtx radioligand binding assays. Separately, α7-nAChR protein complexes isolated from SH-EP1-hα7-Ric-3 and SH-EP1-hα7 were eluted from affinity beads using 1 M carbachol. Eluted proteins were reduced and alkylated before being digested with trypsin in-solution. Digested peptides from each of the five samples prepared from SH-EP1-hα7-Ric-3 and SH-EP1-hα7 cells were analyzed with a Q Exactive mass spectrometer, spectra identified using the Mascot algorithm and results analyzed using ProteoIQ. Identified α7-nAChR associated proteins from SH-EP1-hα7-Ric-3 and SH-EP1-hα7 cells were compared. Associations only identified with Ric-3 co-expression in SH-EP1-hα7-Ric-3 cells were determined to be Ric-3-mediated changes in the α7-nAChR interactome.

α-Bgtx-affinity immobilization was used to isolate α7-nAChR protein complexes from SH-EP1-hα7-Ric-3 and SH-EP1-hα7 cells and associated proteins were identified using mass spectrometry (Fig 1). SH-EP1-hα7-Ric-3 and SH-EP1-hα7 cells provide a robust source of human α7-nAChRs and the differential expression of Ric-3 provides an ideal model in which to investigate the effect of Ric-3 expression on the α7-nAChR interactome. A comparison of α7-nAChR associated proteins in both cell lines allows for the identification of receptor-protein interactions that occur with Ric-3 co-expression. Ric-3-mediated α7-nAChR associated proteins may interact with the receptor during and after direct interaction of Ric-3 with α7-nAChRs. During direct interaction with α7-nAChRs, Ric-3 may recruit other proteins to the receptor complex to facilitate surface expression. After the dissociation of Ric-3, proteins may associate with mature α7-nAChRs as a result of Ric-3-mediated surface expression. The comparison of α7-nAChR complexes from SH-EP1-hα7-Ric-3 and SH-EP1-hα7 cells provides a method of identifying associated proteins, including those that may be essential for Ric-3-mediated enhancement of α7-nAChR surface expression.

## Materials and Methods

### Preparation of α-bgtx-Sepharose affinity beads

Cyanogen bromide-activated Sepharose beads 4B (Sigma-Aldrich, St. Louis, MO) (1 g) were hydrated in 5 mL cold 1mM HCl for 30 minutes and washed with 500 mL 1 mM HCl over a coarse glass filter. The beads were then added to 7.5 mL coupling buffer (0.25 M NaHCO_3_, 0.5 M NaCl, pH 8.3) and subsequently centrifuged at 4°C for 5 minutes at 1,500 x g. The supernatant was discarded, and the pellets were resuspended in 7.5 mL coupling buffer containing 4 mg of α-bgtx (Life Technologies, Eugene, OR). Bead/ligand mixtures were incubated with gentle agitation at 4°C for 18 hours. The beads were subsequently pelleted and resuspended in 7.5 ml of 0.2 M glycine in 80% coupling buffer, 20% ultrapure water and gently agitated overnight at 4°C to block unreacted groups on the beads. The beads were then washed several times over a course glass filter, first with 100 mL of 0.1 M NaHCO_3_, 0.5 M NaCl, pH 8.0, then 100 mL of 0.1 M NaCH_3_CO_2_, 0.5 M NaCl, pH 4.0, again with 100mL of 0.1 M NaHCO_3_, 0.5 M NaCl, pH 8.0, 100 mL coupling buffer, and lastly twice with 100 mL Tris-buffered saline (TBS: 150 mM NaCl, 50 mM Tris, pH 7.5). Washed beads were resuspended in TBS for storage at 4°C. Prior to use, α-bgtx-affinity beads were uniformly resuspended into a slurry and were centrifuged at 4°C for 5 minutes at 1,500 x g. Pelleted beads were resuspended to make a 50/50 slurry with homogenization buffer (100 mM NaCl, 25 mM NaH_2_PO_4_, pH 7.4) before use.

### Cell culture

SH-EP1, SH-EP1-hα7, and SH-EP1-hα7-Ric-3 cells were cultured in DMEM (Sigma-Aldrich, St. Louis, MO) containing 10% horse serum, 5% fetal bovine serum, and 50 μg/ml gentamicin (Life Technologies, Eugene, OR). SH-EP1-hα7 cells were grown with an additional 80 mg/L hygromycin B (Invivogen, San Diego, CA) and SH-EP1-hα7-Ric-3 cells with an additional 80 mg/L hygromycin B and 40 mg/L G418 (Thermo Fisher Scientific Inc., Waltham, MA). The vector used to transfect both cell lines to express human α7-nAChR confers hygromycin B resistance while the vector used to express Ric-3 in SH-EP1-hα7-Ric-3 confers G418 resistance [9, 28]. Cultures were maintained in 75 cm^2^ flasks in a humidified atmosphere containing 5% CO_2_ at 37°C. Cells were processed to isolate solubilized membrane protein when cells were 90% confluent in flasks.

### Membrane protein solubilization

Cells were washed with homogenization buffer (100 mM NaCl, 25 mM NaH_2_PO_4_, pH 7.4) before being mechanically dislodged. Isolated cells were then homogenized with 30 strokes of a Potter-Elvehjem glass homogenizer on ice. SH-EP1-hα7-Ric-3, SH-EP1-hα7, and SH-EP1 membrane solubilization conditions were adapted from Wu, et al. [30]. Membrane fragments were isolated following centrifugation at 10,000 x g for 10 minutes at 4°C. Membrane pellets were then homogenized in solubilization buffer (100 mM NaCl, 25 mM NaH_2_PO_4_, 1% Triton X-100, pH 7.4) with 40 strokes of a Potter-Elvehjem glass homogenizer and incubated for 30 minutes at 4°C with agitation to solubilize membrane-bound proteins. Following centrifugation at 12,600 x g for 10 minutes at 4°C, the solubilized membrane extract was recovered in the supernatant. All buffers used to isolate the solubilized membrane extract were supplemented with protease inhibitors (Roche Applied Science, Indianapolis, IN). Protein content of solubilized membrane extracts was determined using a BCA assay (Pierce).

### Ric-3 immunoblotting

Detergent solubilized receptor preparations (12.5 μg protein per lane) of SH-EP1-hα7-Ric-3 and SH-EP1-hα7 cell lines were used for immunoblotting. Samples were incubated at 60°C for 1 hour with 47.6 mM TCEP and 1x NuPAGE sample buffer (Life Technologies, Eugene, OR), then alkylated in 76.3 mM iodoacetamide at room temperature for 1 hour in the dark. Proteins were separated by SDS-PAGE and transferred at 100 V for 90 minutes to a nitrocellulose membrane (Thermo Fisher Scientific Inc., Waltham, MA). The membrane was blocked in 5% non-fat milk in TBST buffer (150 mM NaCl, 10 mM Tris, 0.05% Tween-20, pH 8) for 1 hour at room temperature and then was incubated with anti-Ric-3 antibodies (ab112911, Abcam, Cambridge, MA) diluted 1:500 in 3% milk TBST buffer overnight at 4°C. After washing with TBST, the membrane was incubated with peroxidase conjugated mouse anti-rabbit secondary antibody (211-032-171, Jackson ImmunoResearch Laboratories, Inc., West Grove, PA) diluted 1:50,000 in 3% milk TBST buffer. The membrane was then washed three times with TBST and twice with TBS (150 mM NaCl, 10 mM Tris, pH 8) before being incubated for 5 minutes in SuperSignal West Pico Chemiluminescent Substrate (Thermo Fisher Scientific Inc., Waltham, MA). Reactive bands were visualized on film after a 3 minute exposure. Ric-3 antibodies were subsequently stripped from blots using Restore Western Blot Stripping Buffer (Thermo Fisher Scientific Inc., Waltham, MA) and probed a second time with anti-GAPDH antibodies diluted 1:1000 in 3% milk TBST buffer overnight at 4°C (14C10, Cell Signaling, Danvers, MA). Following the incubation with anti-GAPDH antibodies, the protocol was the same as described above. Bands were visualized on film after a 30 second exposure.

### α7-nAChR and associated protein complex isolation

Immediately following the isolation of solubilized membrane extracts, a volume containing 3 mg of solubilized protein was incubated with 200 μl of the 50/50 α-bgtx-affinity bead/homogenization buffer slurry for 4 hours at 4°C with gentle agitation. Control samples were solubilized receptor preparations treated with 5 μM MLA (Sigma-Aldrich, St. Louis, MO) during the affinity-immobilization incubation. Following the incubation, α-bgtx-affinity beads and bound protein were transferred to Pierce Spin Cups (Thermo Fisher Scientific Inc., Waltham, MA) and washed several times with solubilization buffer. After washing, the total affinity-immobilized α7-nAChR content was measured using a ^125^I-α-bgtx radioligand binding assay or the isolated proteins were eluted for mass spectrometric analysis (Fig 1).

### Radioligand binding assays

The use of α-bgtx to affinity immobilize α7-nAChRs and concurrently detect them is possible because α7-nAChRs contain multiple α-bgtx binding sites [31]. Affinity-immobilized α7-nAChR content was determined by incubating the membrane protein-α-bgtx-affinity bead complex with 5 nM ^125^I-α-bgtx (Perkin Elmer, Boston, MA) for 1 hour at room temperature. Non-specific binding was determined by the inclusion of 1 μM unlabeled α-bgtx before addition of ^125^I-α-bgtx. Following incubation with ^125^I-α-bgtx, beads were washed three times with solubilization buffer and measured using a Wallac 1275 Minigamma gamma counter.

### Sample preparation, precipitation, and in-solution trypsin digestion

Affinity beads with bound α7-nAChRs and associated proteins were washed three times with solubilization buffer followed by a single high salt solubilization buffer wash (2M NaCl, 25 mM NaH_2_PO_4_, 1% Triton X-100, pH 7.4) to reduce inclusion of non-specific proteins. Immobilized proteins were specifically eluted from the α-bgtx-affinity beads by incubation with 100 μl 1 M carbachol (Sigma-Aldrich, St. Louis, MO) in 20 mM HEPES, pH 8.0 for 30 minutes with agitation every 5 minutes at room temperature. α-Bgtx-affinity beads were allowed to sediment and the eluted proteins in the supernatant were removed and stored at -80°C until preparation for mass spectrometry analysis. Protein content was determined using a Pierce BCA Protein Assay Kit. To prepare for mass spectrometric analysis, samples were thawed and disulfide/sulfhydryl residues were reduced with 47 mM TCEP in 20 mM HEPES, pH 8.0 for 1 hour at 60°C. Samples were alkylated with 83 mM iodoacetamide in 20 mM HEPES, pH 8.0 for 1 hour in the dark at room temperature. Samples were then concentrated and purified via precipitation using a BioRad ReadyPrep 2-D Cleanup Kit (BioRad, Hercules, CA). Precipitated protein was resuspended in 50 mM ammonium bicarbonate, pH 7.8 supplemented with 100 ng trypsin (Promega, Madison, WI) and digested overnight in-solution at 37°C.

### Liquid chromatography & mass spectrometry of protein digests

Tryptic digests were analyzed at the Brown University (Providence, RI) NSF-EPSCoR Proteomics Core Facility with an Agilent 1200 (Agilent Technologies) high performance liquid chromatography (HPLC) in-line with a Q Exactive Hybrid Quadrupole-Orbitrap Mass Spectrometer (Thermo Fisher Scientific Inc., Waltham, MA). Separation of peptides was achieved using a 12 cm Monitor C18 (Column Engineering) reversed-phase column with an internal diameter of 75 μm and integrated 4 μm electrospray ionization tip (self-pack PicoTip, New Objective). Peptides were eluted during a 50 minute linear gradient of 100% solvent A (0.1 M acetic acid in water), 0% solvent B (0.1 M acetic acid in acetonitrile) to 60% solvent A, 40% solvent B) at a flow rate of 200 nl/min and introduced into the mass spectrometer via electrospray ionization (ESI) for analysis (data-dependent mode with 30-second dynamic exclusion with one MS scan followed by nine MS/MS scans). Peak lists of MS/MS spectra were created using msconvert.exe (v. 2.2.3300) available in the ProteoWizard tool [32]. Data were bioinformatically matched against a concatenated target-decoy (sequence-reversed) *Homo sapiens* database (Uniprot, April 2013) using the Mascot algorithm (Matrix Science, Boston, MA). Database searches used the following parameters: Up to two missed trypsin cleaves allowed, 7 ppm MS tolerance, 20 ppm MS/MS tolerance, fixed carbamidomethyl modification, and variable methionine oxidation modification. Mascot search DAT files were loaded into ProteoIQ (Premier Biosoft) for further analysis. Proteins were filtered using a minimum peptide length of 6 amino acids, 1% protein false-discovery rate (FDR) and ≥90% group probability of correct identity assignment using the PROVALT and ProteinProphet algorithm respectively, presence in 2 or more independent replicates, and 0% probability in controls [33–35]. Protein probabilities represent the probability of correct assignment of all observed peptides in a protein group to the identified protein. Both the PROVALT and ProteinProphet algorithm are integrated into ProteoIQ. Only Top and Co-Top identifications, i.e. identifications which include all peptide data in a protein group, were used. Each cell line was analyzed with five biological replicates (Fig 1). Identified proteins were categorized by their reported Gene Ontology (GO) biological process terms using Database for Annotation, Visualization and Integrated Discovery (DAVID) [36]. If an identified protein did not have a GO term for associated biological processes, Protein ANalysis THrough Evolutionary Relationships (PANTHER) was used for classification [37, 38]. If neither classification system had an entry for an identified protein, the protein was classified as unattributed.

## Results

### α-Bgtx-affinity immobilization and ^125^I-α-bgtx radioligand binding assay

**Fig 2:**
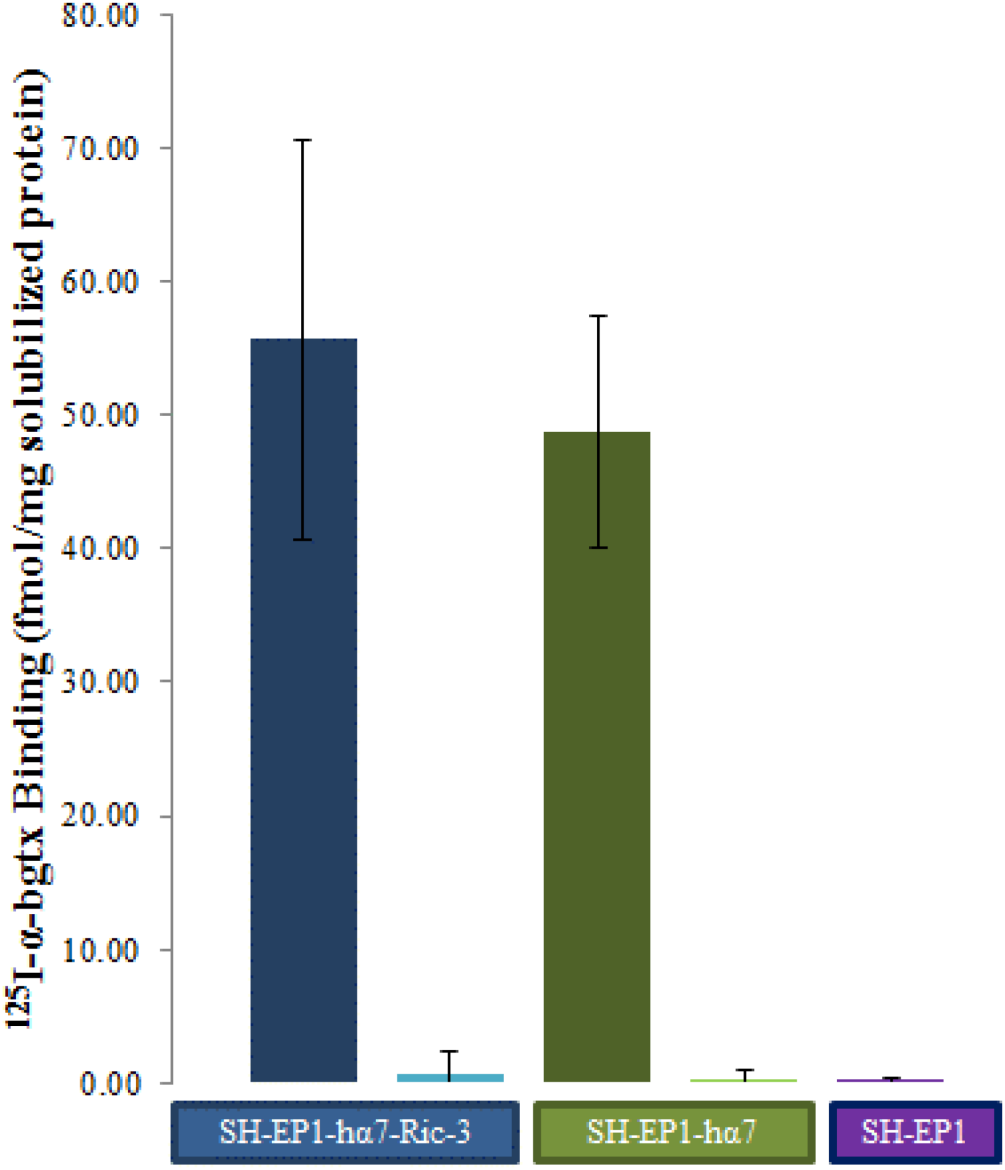
^125^I-α-bgtx binding to affinity immobilized protein. Detergent solubilized membrane extracts were incubated with α-bgtx-affinity beads for 4 hours at 4°C. Protein-α-bgtx-affinity bead complexes were incubated with 5 nM ^125^I-α-bgtx for 1 hour at room temperature. Non-specific binding was determined in controls by the inclusion of 1 μM unlabeled α-bgtx to preparations prior to the addition of ^125^I-α-bgtx. Following incubation with ^125^I-α-bgtx, beads were washed three times with solubilization buffer and measured. Comparable ^125^I-α-bgtx binding activity of protein-α-bgtx-affinity bead complexes isolated from SH-EP1-hα7-Ric-3 (56 ± 15 fmol/mg, in blue) and SH-EP1-hα7 (49 ± 9 fmol/mg, in green) was observed (Student’s *t* test, p = 0.40) while SH-EP1 preparations (in purple) did not show α-bgtx binding activity. No ^125^I-α-bgtx binding to protein-α-bgtx-affinity bead complexes was observed in samples treated with 5 μM MLA confirming α7-nAChR specificity (Student *t* test, p < 0.05). SH-EP1-hα7-Ric-3 and SH-EP1-hα7 ^125^I-bgtx binding activity was analyzed with five independent biological replicates. MLA treated samples and SH-EP1 ^125^I-α-bgtx binding activity were analyzed with three independent biological replicates.

^125^I-α-bgtx binding assays were used to determine levels of α7-nAChR content isolated on α-bgtx affinity beads. Solubilized membrane extracts from SH-EP1-hα7-Ric-3 and SH-EP1-hα7 cell lines were incubated with α-bgtx-affinity beads to isolate α7-nAChRs for further analysis. Comparable ^125^I-α-bgtx binding levels were observed for α-bgtx-affinity immobilized protein from both SH-EP1-hα7-Ric-3 and SH-EP1-hα7 cell solubilized receptor preparations (56 ± 15 and 49 ± 9 respectively fmol ^125^I-α-bgtx/mg solubilized protein) (Fig 2). Consistent with published reports, ^125^I-α-bgtx binding was undetectable in untransfected SH-EP1 cells [26, 28]. Additionally, in SH-EP1-hα7-Ric-3 and SH-EP1-hα7 cell preparations, ^125^I-α-bgtx binding was reduced by more than 99% by the addition of 5 μM MLA, a selective, high-affinity ligand of the α7-nAChR. This result provides evidence that the α7-nAChR is the principal α-bgtx-sensitive protein isolated on α-bgtx-affinity beads (Fig 2).

### Immunoblot confirmation of Ric-3 expression

**Fig 3:**
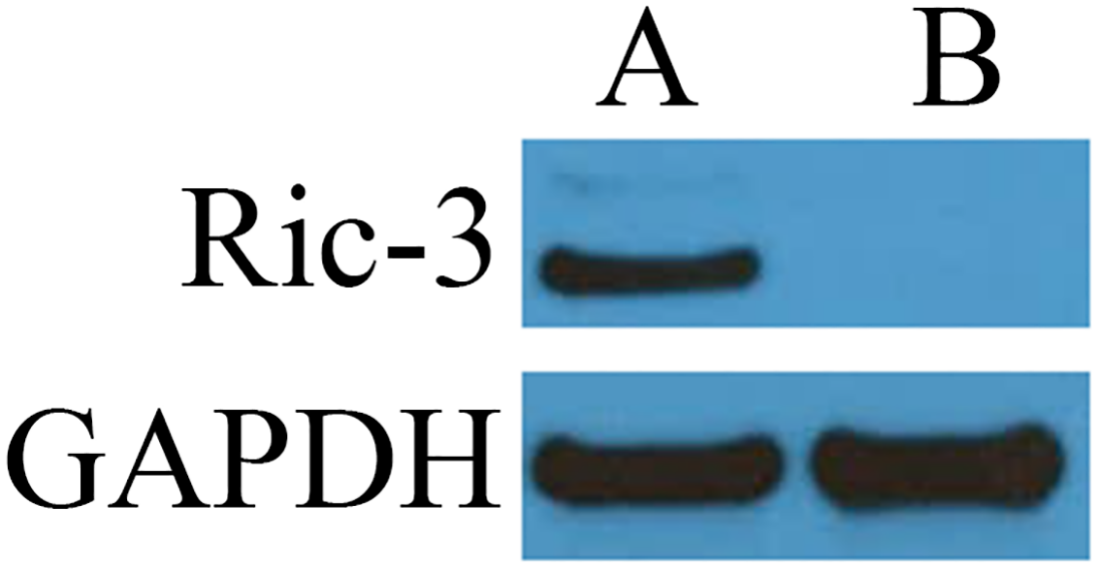
Ric-3 immunoreactivity in SH-EP1-ha7-Ric-3. Solubilized membrane extracts of SH-EP1-hα7-Ric-3 and SH-EP1-hα7 cell lines were probed with anti-Ric-3 polyclonal antibodies. Ric-3 antibody immunoreactivity at 41 kDa confirms the presence of Ric-3 in membrane extracts from SH-EP1-hα7-Ric-3 cells (A). There is no corresponding band in SH-EP1-hα7 membrane extracts (B). The anti-GAPDH antibody immunoreactivity was utilized as a loading control.

SH-EP1 cells have been shown previously to lack detectable protein levels of the chaperone Ric-3. Immunoblotting was used to probe Ric-3 immunoreactivity in solubilized receptor preparations of SH-EP1-hα7-Ric-3 and SH-EP1-hα7 cells. Solubilized receptor preparations from SH-EP1-hα7-Ric-3 cells contain Ric-3 immunoreactivity while no immunoreactivity was observed in preparations of SH-EP1-hα7 cells (Fig 3).

### α7-nAChR associated proteins

Carbachol elutions of α-bgtx-affinity immobilized α7-nAChRs from both SH-EP1-hα7-Ric-3 and SH-EP1-hα7 cell preparations were analyzed using a Q Exactive Hybrid Quadrupole-Orbitrap Mass Spectrometer (Thermo Fisher Scientific Inc., Waltham, MA). We set the following *a priori* inclusion criteria parameters to identify proteins: 1% protein FDR, ≥ 90% group probability of correct identity assignment, and the presence in two or more of five independent biological replicates with 0% probability of correct identity assignment in controls as determined by the ProteinProphet algorithm. Using these criteria, the α7-nAChR was identified in all SH-EP1-hα7-Ric-3 and SH-EP1-hα7 cell replicates with 100% and 98% probability, respectively, by way of the peptide FPDGQIWKPDILLYNSADER (Table 1). The identified α7-nAChR peptide was not identified as a peptide from the reported sequence of the CHRFAM7A protein product (Accession number Q494W8). Ric-3 was detected in SH-EP1-hα7-Ric-3 cell samples and met all inclusion criteria, but it was associated with a borderline probability score of 88% (compared to the preset criteria of ≥ 90%). This may reflect the fact that Ric-3 is only transiently associated with α7-nAChRs. Not all α7-nAChRs will be interacting with Ric-3 at the time of α-bgtx-affinity bead isolation. The 2 M NaCl washes for all samples were also analyzed to confirm that α7-nAChRs were not eluted during the washing of material bound to α-bgtx-affinity beads. Neither α7-nAChR peptides nor Ric-3 peptides were identified in the 2 M NaCl bead wash from SH-EP1-hα7-Ric-3 cell samples. Identification of the α7-nAChR in SH-EP1-hα7-Ric-3 and SH-EP1-hα7 cell samples confirms that α7-nAChRs were eluted from the α-bgtx-affinity beads by the cholinergic agonist carbachol. Neither α7-nAChR nor Ric-3 peptides were identified in carbachol-eluted samples prepared from SH-EP1 cells, which lack expression of both proteins.

**Table 1:** Identification of α7-nAChR in SH-EP1-hα7-Ric-3 and SH-EP1-hα7 cells.

Protein name	Accession number	Cell line	Total peptides	Sequence coverage (%)	Data sets	Probability score (%)
Neuronal acetylcholine receptor subunit alpha-7	P36544	SH-EP1-hα7-Ric-3	1	3.98	5	100
	P36544	SH-EP1-hα7	1	3.98	5	98

A peptide corresponding to α7-nAChR subunits was identified in α-bgtx-enriched samples of both SH-EP1-hα7-Ric-3 and SH-EP1-hα7 cell lines. Data analysis was performed using ProteoIQ version 2.7 Protein inclusion criteria include 1% protein FDR, minimum peptide length of six amino acids ≥90% probability, identification in 2 or more of 5 replicates (i.e., Data sets), and 0% probability in controls. FDRs were determined using the PROVALT algorithm and probabilities were determined with the ProteinProphet algorithm through ProteoIQ analysis. Only Top and Co-Top identifications were considered.

Proteins identified in our analysis of the α7-nAChR interactome are most likely components of large protein complexes and may either be associating directly with the receptor or with another member of the complex. Comparison of carbachol-eluted proteins from SH-EP1-hα7-Ric-3 and SH-EP1-hα7 identified thirty-nine Ric-3-promoted α7-nAChR associated proteins (Table 2). Fourteen of the thirty-nine proteins identified as Ric-3-mediated have previously been reported as associated with a cellular process known to affect protein expression (Table 2, category labeled blue). These fourteen Ric-3-mediated associated proteins may be directly or indirectly recruited by Ric-3 to facilitate receptor assembly and targeting. In addition to proteins associated with protein expression, seven proteins are associated with protein turnover, four with signaling, and fourteen associated with other processes (Table 2). In total, seven of the thirty-nine proteins have functions previously shown to affect nAChRs (Table 3). Six of the thirty-nine proteins listed in Table 2 were identified with a single unique peptide in two or more replicates and are summarized in Table 4. Peptide-level detail for all thirty-nine proteins is provided in S1 Table.

**Table 2:** Ontological grouping of Ric-3-mediated α7-nAChR associated proteins.

Biological process	Associated proteins	Accession number	Total peptides	Seq. cov. (%)	Data sets	Prob. score (%)	Category
Apoptotic process	KN motif and ankyrin repeat domain-containing protein 2	Q63ZY3	4	6.6	3	100	Protein turnover
	Tax1-binding protein 1	Q86VP1	5	7.5	3	100	Protein turnover
Cell cycle	Cell cycle progression protein 1	Q9ULG6	5	11.3	3	99	Signaling
Cytoskeletal organization	Rho guanine nucleotide exchange factor 17	Q96PE2	1	0.7	2	98	Signaling
	SUN domain-containing protein 2	Q9UH99	3	6.4	2	95	Other proteins
Developmental process (Developmental process, regulation of "*")	Keratin, type I cytoskeletal 15	P19012	11	26.8	5	100	Other proteins
	Keratin, type II cuticular Hb4*	Q9NSB2	6	11.5	5	96	Other proteins
	Keratin, type II cytoskeletal 75 (P)	O95678	15	25.1	5	100	Other proteins
Ion transport	Ferritin light chain	P02792	3	24.6	2	100	Other proteins
Nucleobase, nucleoside, nucleotide, and nucleic acid metabolic process	5'-nucleotidase	P21589	6	18.1	3	100	Other proteins
	FAD synthase	Q8NFF5	2	7.4	2	98	Other proteins
	Nuclear receptor coactivator 4	Q13772	3	5.7	2	100	Protein turnover
	TRMT1-like protein	Q7Z2T5	1	2.1	3	90	Other proteins
Protein complex assembly	Erythrocyte band 7 integral membrane protein	P27105	2	10.1	2	99	Other proteins
	Gamma-adducin (P)	Q9UEY8	5	8.8	3	100	Surface expression
Protein folding	Calnexin	P27824	8	16.1	5	100	Surface expression
	Calreticulin	P27797	6	25.4	3	100	Surface expression
	DnaJ homolog subfamily B member 11	Q9UBS4	2	6.7	3	93	Surface expression
	Peptidyl-prolyl cis-trans isomerase A	P62937	4	32.7	4	98	Surface expression
	T-complex protein 1 subunit epsilon	P48643	3	9.1	2	93	Surface expression
Protein transport	ADP-ribosylation factor 4	P18085	3	20.0	2	95	Surface expression
	Autophagy-related protein 9A	Q7Z3C6	1	2.3	2	91	Protein turnover
	Optineurin	Q96CV9	1	2.3	2	96	Surface expression
	Translocon-associated protein subunit gamma	Q9UNL2	1	7.6	2	99	Surface expression
Protein Modification	Dolichol-phosphate mannosyltransferase	O60762	7	32.7	5	91	Surface expression
	LIM domain only protein 7	Q8WWI1	2	1.5	2	94	Protein turnover
	Tyrosine-protein phosphatase non-receptor type 14	Q15678	2	4.2	3	92	Surface expression
	Ubiquitin-like modifier-activating enzyme 1	P22314	4	6.3	2	100	Protein turnover
Regulation of biosynthetic process	Protein LYRIC	Q86UE4	2	9.3	2	99	Other proteins
Response to stress	Hypoxia up-regulated protein 1	Q9Y4L1	7	10.3	3	100	Surface expression
	Calcium-binding and coiled-coil domain-containing protein 2	Q13137	8	23.5	5	100	Protein turnover
	Peroxidasin homolog	Q92626	9	8.5	5	100	Other proteins
Signal Transduction	Angiopoietin-related protein 2	Q9UKU9	3	7.5	2	99	Signaling
	cAMP-dependent protein kinase type I-alpha regulatory subunit	P10644	2	8.4	2	92	Surface expression
	Inositol 1,4,5-trisphosphate receptor type 1	Q14643	4	1.8	2	90	Signaling
	Reticulocalbin-3 (P)	Q96D15	1	3.7	2	98	Surface expression
Unattributed	BTB/POZ domain-containing protein 2	Q9BX70	4	11.1	4	100	Other proteins
	RNA-binding protein 33	Q96EV2	4	4.4	2	92	Other proteins
	Uncharacterized protein	F5H7S3	7	24.0	3	97	Other proteins

**Table 3:** Summary analysis of Ric-3-mediated proteins with literature citations implicating functional interactions with nAChRs.

Protein Summary	Accession number	Citation type	α7-nAChR only	α7 and other nAChRs	non-α7 nAChRs only
Calnexin	P27824	Specific Protein			[45–58]
Calreticulin	P27797	Specific Protein			[45–48]
cAMP-dependent protein kinase type I-alpha regulatory subunit	P10644	Both		[63,64,66–70]	
Dolichol-phosphate mannosyltransferase	O60762	Protein Class		[3, 42–45]	
Inositol 1,4,5-trisphosphate receptor type 1	Q14643	Both		[82–85]	
Peptidyl-prolyl cis-trans isomerase A	P62937	Protein Class	[45, 49]		
Tyrosine-protein phosphatase non-receptor type 14	Q15678	Protein Class		[6,59,61,62]	

**Table 4:** Summary of single-peptide-based protein identifications.

Protein Summary	Accession number	m/z	z	Peptide sequence	Score	Replicate
Autophagy-related protein 9A	Q7Z3C6	889.85	2	ESDESGESAPDEGGEGAR	42.29	4
	Q7Z3C6	889.85	2	ESDESGESAPDEGGEGAR	57.04	5
Optineurin	Q96CV9	719.83	2	SEIETQTEGSTEK	20.43	4
	Q96CV9	719.83	2	SEIETQTEGSTEK	77.71	5
Reticulcalbin-3	Q96D15	633.28	2	VADQDGDSMATR	87.64	1
	Q96D15	633.27	2	VADQDGDSMATR	85.03	2
Rho guanine nucleotide exchange factor 17	Q96PE2	640.81	2	LSSGGGSSSETVGR	89.08	4
	Q96PE2	640.81	2	LSSGGGSSSETVGR	89.33	5
Translocon-associated protein subunit gamma	Q9UNL2	854.42	2	QQSEEDLLLQDFSR	109.45	4
	Q9UNL2	854.41	2	QQSEEDLLLQDFSR	19.33	5
TRMT1-like protein	Q7Z2T5	822.37	2	TTDDTTTDNYIAQGK	58.93	2
	Q7Z2T5	822.37	2	TTDDTTTDNYIAQGK	57.62	4
	Q7Z2T5	822.37	2	TTDDTTTDNYIAQGK	54.36	5

Receptor-α-bgtx bead complexes were eluted with carbachol from α-bgtx-affinity resin, reduced, alkylated, precipitated and digested with trypsin in solution. Tryptic peptides were then analyzed using a Q Exactive mass spectrometer. Thirty nine Ric-3 stimulated proteins were identified through comparison of carbachol-eluted proteins from SH-EP1-hα7-Ric-3 and SH-EP1-hα7 α-bgtx-affinity immobilized samples. Each condition was analyzed with five replicates (i.e., Data sets). Data analysis was performed using ProteoIQ version 2.7. Protein inclusion criteria included 1% protein FDR, minimum peptide length of six amino acids, ≥90% probability, identification in 2 or more of 5 replicates, and 0% probability in controls. FDRs were determined using the PROVALT algorithm and probabilities were determined with the ProteinProphet algorithm through ProteoIQ analysis. Only Top and Co-Top identifications were considered. Biological processes are listed as determined by DAVID analysis of Gene Ontology (GO) terms. Biological process GO terms for six proteins are not available. Biological processes for three of these proteins were available through PANTHER analysis (denoted by “(P)”) and the remaining three are listed as unattributed. Each protein listed is categorized as potentially involved with surface expression, protein turnover, signaling, or associated with biological processes not included in the previous categories as “Other proteins”.

Seven of the thirty-nine proteins identified as Ric-3-mediated have reported functions which have been previously shown to affect nAChRs. The cited literature reporting the relationships between each of the listed proteins and nAChRs is categorized as either indicating a direct association to the listed proteins specifically, or linked by a previously associated class of proteins (e.g., tyrosine phosphatases), or by both. Two proteins, cAMP-dependent protein kinase type I-alpha regulatory subunit and inositol 1,4,5-trisphosphate receptor (IP_3_R) type 1 are listed as both since the literature does not always distinguish either specific proteins in the PKA complex nor which IP_3_R type is being discussed. These proteins are further separated by whether their functions have been associated with α7-nAChRs only, other nAChR subtypes but not α7-nAChRs or with α7 and other nAChR subtypes.

Six proteins from Table 2 were identified by one unique peptide. Mass to charge ratios (m/z), charges (z), peptide sequence, and Mascot ion scores are listed for each single-peptide-based identification. For analysis, replicates were assigned replicate numbers one through five. Each identification is listed separately for each replicate number in which the single-peptide was observed.

Ninety-seven proteins were uniquely isolated on α-bgtx-affinity beads from SH-EP1-α7 cells that were not identified in preparations from SH-EP1-hα7-Ric-3 cells. These proteins represent possible protein associations with α7-nAChRs that form in the absence of Ric-3 expression (S2 Table). A total of 625 proteins that met the inclusion criteria were identified in both cell lines (S3 Table). These proteins common to both cell lines may represent general α7-nAChR interacting proteins or non-specific interactions with α-bgtx-affinity beads. Analysis of the cellular compartment GO terms for proteins unique to SH-EP1-hα7-Ric-3 samples and those unique to SH-EP1-α7 samples suggests a difference in cellular distribution of the receptors between the two cell lines (S4 Table). The reported Ric-3-mediated interactome consists of proteins associated with the cytosol, intracellular membranes, and the ER. Many of the identified Ric-3-mediated proteins are reported to be localized in the ER, which agrees with previous reports that Ric-3 is a chaperone predominantly expressed in the ER. In comparison, none of the proteins identified as unique in SH-EP1-α7 samples have been reported to be localized in the ER.

## Discussion

To identify the Ric-3-mediated α7-nAChR interactome, specific α-bgtx-binding proteins were isolated from cells stably expressing the receptor alone or the receptor and Ric-3 using α-bgtx affinity beads and specifically eluted using a cholinergic agonist. Eluted proteins were digested with trypsin and the resulting peptides were analyzed with mass spectrometry. Analysis of peptide fragmentation spectrum was used to identify the proteins that associated with α7-nAChRs in samples isolated from cells expressing, or not expressing Ric-3. Identified in this study were thirty-nine proteins whose association with α7-nAChR was mediated by co-expression of Ric-3.

### Cellular model

The two cell lines utilized, SH-EP1-hα7-Ric-3 and SH-EP1-hα7, heterologously express human α7-nAChRs and differentially express the chaperone Ric-3. These cell lines were chosen to study the Ric-3-mediated α7-nAChR interactome for several reasons. First, the use of these two transfected cell lines provides a level of control for selective expression of the two proteins of interest that would be more difficult to achieve using endogenous expression models. Second, these two cell lines are a reliable source of α7-nAChR and Ric-3 expression.

Previously, fifty-five α7-nAChR interacting proteins were identified by tandem mass spectrometry by comparison of α-bgtx affinity immobilized protein from α7-nAChR wild type and α7-nAChR knockout mouse brain tissue [29]. However, α7-nAChR peptides were not identified by tandem mass spectrometry in this study. Although the α7-nAChR was identified in the study presented here, none of the fifty-five α7-nAChR interacting proteins identified in the previous study were identified. In addition to the important distinction that we identified the α7-nAChR while the previous study did not, there are several differences between the present study and the previous study that may account for the disparity between the two identified interactomes. Substantial modifications were made to the α-bgtx-affinity immobilization protocol and mass spectrometry sample preparation in order to maximize isolation and detection of α7-nAChRs. The model system in the investigation presented here is also human in origin and used clonal cells of a single morphology as compared to the heterogeneity of the cell types found in of the murine brain. The work shown here investigates a more focused, Ric-3-mediated α7-nAChR interactome, rather than a general α7-nAChR interactome, which was the aim of the previous study.

### Ric-3-mediated α7-nAChR associated proteins

**Fig 4:**
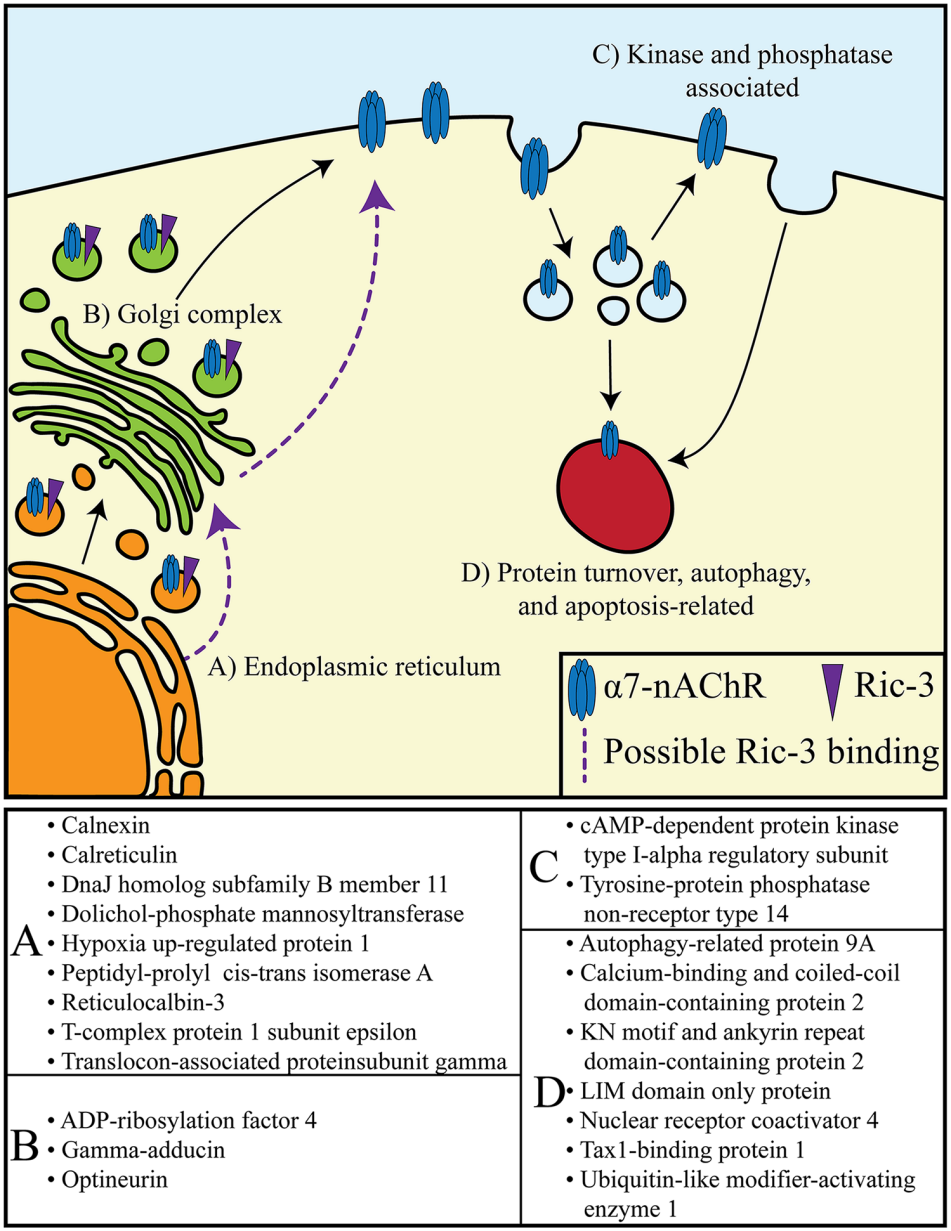
Proteins that could affect the life-cycle of α7-nAChRs. A total of twenty-one identified proteins have functions that could affect the life-cycle of the α7-nAChR, e.g., receptor biogenesis, modulation of intracellular and plasma-membrane expressed receptor pools, as well as receptor turnover, autophagy, or apoptosis related. These proteins are grouped based on their reported cellular compartment localization. The activity of these proteins may be localized to the endoplasmic reticulum (A), the Golgi complex (B), or the cytosol (C&D). Cytosolic proteins can either be involved in the mobilization of internal pools of α7-nAChRs through kinase and phosphatase activity (C) or be associated with protein turnover, autophagy, and apoptosis-related processes (D).

The role of the molecular chaperone Ric-3 in α7-nAChR expression has been investigated by a number of different methods in multiple models and previous reports have demonstrated an increase in cell surface expression of α7-nAChRs in cells also expressing Ric-3 [9, 23, 26, 39]. Human cells lines were used to identify α7-nAChR protein-associations that appear with co-expression of Ric-3. Of a total of thirty-nine identified members of the Ric-3-mediated α7-nAChR interactome, fourteen proteins have been previously reported to be associated with a process known to affect protein expression. Of the remaining proteins, five are associated with signal transduction/intracellular signaling, seven with protein catabolism and/or autophagy, and fourteen that do not have a reported connection to α7-nAChR surface expression, signaling, protein catabolism or autophagy (Table 2). The fourteen proteins associated with protein expression as well as the seven proteins associated with protein catabolism and/or autophagy may represent receptor-protein interactions contributing to the life-cycle of α7-nAChRs (Fig 4). Ric-3 was identified by mass spectrometry with 88% probability (versus the *a priori* 90% inclusion criteria) and met all other inclusion criteria. The probability of correct identification of Ric-3 may fall outside the preset inclusion criteria due to its transient interaction (interacting intracellularly) with α7-nAChR. That transient interaction of Ric-3 nevertheless may lead to the interactions with the α7-nAChR protein complex identified in this study.

### Endoplasmic reticulum localized associated proteins

Evidence suggests that two of the Ric-3-mediated α7-nAChR-associated proteins are involved in early stages of protein expression in the ER. First, translocon-associated protein subunit gamma is a TRAP protein which interacts with SEC61 and is involved in protein translocation in the ER [40, 41]. Second, dolichol-phosphate mannosyltransferase, is an enzyme that may be involved in N-glycosylation (Table 3). N-glycosylation and subsequent glucose trimming is an important regulatory step in protein expression in the ER, and α7-nAChRs have been shown to be glycosylated [3, 42–45]. Further investigation is required to deduce if dolichol-phosphate mannosyltransferase may be involved with α7-nAChR N-glycosylation. In addition, seven proteins associated with protein folding and receptor assembly were identified: calnexin; calreticulin; peptidyl-prolyl cis-trans isomerase A; DnaJ homolog subfamily B member 11; hypoxia up-regulated protein 1; t-complex protein 1 subunit epsilon; and reticulocalbin-3.

Calnexin and calreticulin are two ER chaperones which bind to unfolded or misfolded proteins and are central to a cycle of repeated folding and unfolding [45]. The calnexin/calreticulin cycle is a well-studied ER mechanism for achieving proper protein folding and receptor assembly. The calnexin/calreticulin cycle has also been identified previously as important for muscle nAChR localization (Table 3) [45–48]. However, the interaction of both chaperones with α7-nAChRs has not been previously reported. In addition to the two chaperones, a number of other proteins have been shown to have a role in the calnexin/calreticulin cycle. Peptidyl-proyl cis-trans isomerases such as peptidyl-prolyl cis-trans isomerase A may also contribute to the calnexin/calreticulin cycle and have been shown to enhance α7-nAChR folding in the ER (Table 3) [45, 49]. Moreover, BiP, another chaperone associated with protein expression, has been previously shown to associate with α subunits of the muscle type nAChR [50–52]. BiP is a member of a large ER protein complex, and while BiP itself was not identified as a α7-nAChR-associated protein in this study, two other members of the BiP complex were identified: DnaJ homolog subfamily B member 11 and hypoxia up-regulated protein 1 [53]. The identification of DnaJ homolog subfamily B member 11 and hypoxia up-regulated protein 1 as proteins in complex with α7-nAChR suggests the possible involvement of the BiP complex in facilitating protein folding in the ER. The interaction of muscle-type nAChR subunits with BiP is short lived [48]. If the interaction with α7 subunits is similarly short lived, BiP itself would not be identified in this study. T-complex protein 1 subunit epsilon is a member of the BBS/CCT complex which facilitates protein folding through a complex mechanism of trapping unfolded proteins that undergo a series of ATP hydrolysis-driven confirmation changes to induce proper folding [54]. CCT complexes have been associated previously with a myriad of proteins but not with nicotinic subunits [55]. Additionally, reticulocalbin-3 is a calcium binding protein localized to the ER and has been shown to facilitate maturation of certain proteins. Based on its identification in the current study, reticulocalbin-3 may have a similar function in the biosynthesis of α7-nAChRs [56, 57].

### Associated proteins localized in the Golgi complex

Following proper folding and receptor assembly, nicotinic receptors are transported to the Golgi complex before being transported to the cell surface. Once at the plasma membrane, receptors may undergo endocytosis to be recycled to the Golgi complex, recycled back to the plasma membrane, or be degraded. Three proteins that were identified as regulated through Ric-3 in SH-EP1-hα7-Ric-3 cells are associated with protein trafficking. Gamma-adducin is a membrane-cytoskeleton-associated protein that promotes protein exit from the Golgi complex by remodeling the actin network surrounding the Golgi complex. Optineurin is a protein vital to the maintenance of Golgi complex structure in addition to being implicated in trafficking from the Golgi complex to the plasma membrane [45]. ADP-ribosylation factor 4 is associated with recycling proteins from endosomes to the trans-Golgi network [58].

### Identified kinase and phosphatase associated proteins

Both kinase and phosphatase activity has been implicated in nAChR up-regulation [6, 59, 60]. One kinase subunit and one phosphatase were identified: cAMP-dependent protein kinase type I-alpha regulatory subunit and tyrosine-protein phosphatase non-receptor type 14. Tyrosine dephosphorylation has been shown to increase α7-nAChR surface expression in oocytes by promoting exocytosis of intracellular receptor pools (Table 3) [59, 61]. Conversely, tyrosine phosphatase activity has been shown to promote muscle-type nAChR turnover, emphasizing how nAChR subtypes may respond differently to the same modification [6, 62]. Kinase activity of cAMP-dependent protein kinase (PKA) has been shown to increase α7-nAChR surface expression in neonatal rat sympathetic neurons as well as in human embryonal kidney cells [63, 64]. PKA enzymes are comprised of four subunits, two catalytic and two regulatory [65]. The cAMP-dependent protein kinase type I-alpha regulatory subunit has previously been shown to colocalize with cholinergic markers [66]. Activation of α7-nAChRs has also been shown to stimulate PKA activity (Table 3) [67]. The identification of cAMP-dependent protein kinase type I-alpha regulatory subunit, coupled with these previous observations suggest that PKA activity may be linked to α7-nAChRs through the association of one of the enzyme’s regulatory subunits. PKA activity in turn may have a diverse effect through other pathways leading to numerous biological processes, such as enhancement of synaptic efficiency and nicotine-stimulated long term potentiation [68–70]. Determining whether the effects of kinases and phosphatases are through direct phosphorylation or dephosphorylation of nAChRs or effects upon a member of the nAChR interactome requires additional study. There may also be a temporal component with phosphorylation or dephosphorylation occurring at different stages of nAChR biogenesis.

### Protein turnover, autophagy and apoptosis related proteins

Many of the mechanisms and pathways that are utilized in receptor turnover may overlap with other mechanisms such as autophagy [71]. Seven of the Ric-3-mediated α7-nAChR-associated proteins identified have been reported to play a role in receptor turnover, apoptosis or autophagy: nuclear receptor coactivator 4, autophagy-related protein 9A, ubiquitin-like modifier-activating enzyme 1, LIM domain only protein 7, calcium-binding and coiled-coil domain-containing protein 2, KN motif and ankyrin repeat domain-containing protein 2, and tax1-binding protein 1 [72–81]. Several mechanisms may regulate the association of autophagy with α7-nAChR only when Ric-3 is expressed. The associated proteins could also be involved in other pathways related to autophagy, such as protein catabolism [71]. In theory, with Ric-3 co-expression, more α7-nAChRs reach the surface of the cell and are subject to mechanisms regulating receptor turnover. In cells in which dramatically fewer α7-nAChRs reach the cell surface (i.e., cells in which Ric-3 is not co-expressed), the proteins involved with such turnover functions would be diminished as well.

### Signal transduction and intracellular signaling associated proteins

In addition to the surface expression-related proteins described above, Ric-3 co-expression appears to enhance association of α7-nAChRs with proteins involved in signal transduction and intracellular signaling. These include: Inositol 1, 4, 5-trisphosphate receptor type 1; cell cycle progression protein 1; Rho guanine nucleotide exchange factor 17; and angiopoietin-related protein 2. These interactions are of interest because Ric-3-mediated co-expression may promote subsequent signaling cascades. Inositol 1, 4, 5-trisphosphate receptor (IP_3_R) type 1 is associated with intracellular Ca^2+^ release and signaling. Nicotine stimulation of α7-nAChRs has been shown to lead to Ca^2+^ flux through IP_3_R type 1 and through activation of phospholipase C (PLC) (Table 3) [82–85]. In addition to the effect of α7-nAChRs on the activity of IP_3_Rs, it has also been shown that α7-nAChRs colocalize with IP_3_Rs in PC12 cells [85]. IP_3_Rs have also been shown to colocalize with muscle-type nAChRs at the neuromuscular junction in rat skeletal muscle [86]. Expanding on what was shown previously, the association of IP_3_Rs with α7-nAChR identified in this study reflects a direct interaction between the IP_3_R and the α7-nAChR interactome. Cell cycle progression protein 1, Rho guanine nucleotide exchange factor 17, and angiopoietin-related protein 2 are associated with RhoA GTPases and may be involved in a number of processes [87–89]. Angiopoietin-related protein 2, for example, has been linked to cellular processes such as angiogenesis and cell motility, and members of the small RhoA GTPase family participate in the endocytosis of muscle-type nAChRs [90–95].

### Additional identified associated proteins

Fourteen proteins were identified that are not currently linked to α7-nAChR surface expression, signal transduction/intracellular signaling, or protein catabolism and/or autophagy. These fourteen proteins are associated with the cytoskeletal organization; developmental processes; ion transport; nucleobase, nucleoside, nucleotide, and nucleic acid metabolic processes; biosynthetic processes; response to stress, or do not currently have a well-defined associated biological process. Several of the additional proteins identified are associated with the cytoskeleton and developmental processes (i.e., SUN domain-containing protein 2; keratin, type I cytoskeletal 15; keratin, type II cuticular Hb4; keratin, and type II cytoskeletal 75) and may be involved in the subcellular localization of nAChRs. Keratin however is often considered to be non-specific contaminant in mass spectrometry investigations and these proteins would have to be investigated further to confirm specificity to α7-nAChRs. Ferritin light chain is a subunit of the protein ferritin, which is involved in the transport of iron [96]. Ferritin light chain was shown previously to be enriched in autophagosomal fractions from cancer cell lines as was calcium-binding and coiled-coil domain-containing protein 2, optineurin, autophagy-related protein 9A, all of which were also identified in this study [76]. Several identified proteins were associated with nucleobase, nucleoside, nucleotide, and nucleic acid metabolic processes: 5'-nucleotidase, FAD synthase, and TRMT1-like protein. Of these three proteins, 5’-nucleotidase is of interest as it is a marker for types of lipid rafts and during hypoxia is involved with nAChR-simulated adenosine production [97, 98]. The biological process of Erythrocyte band 7 integral membrane protein was characterized by DAVID as protein complex assembly though this attribution refers to the proteins ability to form homo-oligomers [99]. Erythrocyte band 7 integral membrane protein is of particular interest due to its previous association with lipid rafts and possible regulation of ion channel activity [100]. The protein LYRIC is a marker found in numerous cancer cell lines [101, 102]. Peroxidasin homolog is an extracellular matrix component that may be associated with reactive oxygen species metabolism [103, 104]. There is currently no literature reporting on the biological processes of BTB/POZ domain-containing protein 2, RNA-binding protein 33, and uncharacterized protein “TPM1” (Uniprot Accession F5H7S3). These proteins represent a population with an assortment of different biological functions that warrant further investigation to discern the functionality of their relationship with α7-nAChR.

## Conclusions

Receptor-protein interactions are dynamic and dependent upon many factors. Identifying α7-nAChR-associating proteins as described in this study captures a snapshot of possible interactions under standard tissue culture conditions. A single peptide of the human α7-nAChR subunit was detected in all SH-EP1-hα7-Ric-3 and SH-EP1-hα7 samples. This reproducibly identified single α7-nAChR subunit peptide would be ideal for absolute quantitation using mass spectrometry that may be of interest for future studies investigating α7-nAChR expression.

SH-EP1-hα7-Ric-3 and SH-EP1-hα7 cells are ideal for identifying Ric-3-mediated α7-nAChR-associating proteins though it is possible that in this model, interactions are present that would not occur endogenously in native cells. It is therefore important to develop appropriate methodologies to continue these investigations in models that endogenously express α7-nAChRs, such as SH-SY5Y cells [27]. Additional protein interactions with α7-nAChRs and other nAChR subtypes have been reported by other groups that were not identified in this study [5, 105]. Our inability to detect these previously identified α7-nAChR-associated proteins may reflect the ability of some proteins to compete with α-bgtx binding, and thus be affected during the α-bgtx affinity bead incubation. For example, the three-fingered toxin family member Lynx1 (aka SLURP2) has been shown to interact competitively with α-bgtx for binding to α7-nAChRs and was not identified in this study [106, 107].

This investigation expands upon our previous work from a murine model to a human model of α7-nAChR-associating proteins. The work described here is an example of how α-bgtx-affinity may be harnessed as a tool for proteomic investigations of α7-nAChRs. Here we investigated receptor-protein interactions mediated by the differential expression of the Ric-3 chaperone. This approach can be applied to any protein to investigate possible alterations on the α7-nAChR interactome. Furthermore, this approach reproducibly identified a tryptic peptide of the α7-nAChR subunit. This peptide was identified in all SH-EP1-hα7-Ric-3 and SH-EP1-hα7 samples and was not observed in SH-EP1 samples. The size and reproducibility of this peptide could be used for absolute quantitation of α7-nAChRs by mass spectrometry using a heavy-labeled variant of the peptide. The study reported here presents a unique investigation of the role of Ric-3 in modification of the proteins associating with α7-nAChR. Identifying these proteins as members of the α7-nAChR macromolecular complex provides vital insight for understanding α7-nAChR surface expression and may assist in the identification of future therapeutic targets.

## Supporting Information

S1 Fig Uncropped Fig 3 Ric-3 and GAPDH immunoblots. Immunoblots for investigation of Ric-3 and GAPDH immunoreactivity in SH-EP1-hα7-Ric-3 (A) and SH-EP1-hα7 (B) solubilized membrane extracts. Blots are initially probed for Ric-3 immunoreactivity, stripped, and subsequently probed again for GAPDH immunoreactivity to confirm consistent gel loading.
(TIF)

S1 Table Characterization of each peptide identified to infer the identity of the 39 Ric-3-promoted α7-nAChR-associated proteins reported in Table 2. “Group number” lists all proteins, 1 through 39, in the order in which they were grouped by ProteoIQ during analysis. Only Top and Co-Top protein identifications, i.e. only proteins identifications that can account for all peptide information within a protein group, were analyzed. For all 39 identified proteins, all Top and Co-Top identifications were either different isoform entries for protein products of the same gene or alternative database entries. Uniprot accession numbers, protein names, and gene names are provided for each Top and Co-Top entry. Also described per Top and Co-Top entry are probability score, protein score, the number of unique peptides identified, and sequence coverage. The observed mass to charge (m/z), charge (z), ion score, peptide sequence, and possible modifications are listed for every peptide identified for both Top and Co-Top entries. For analysis, replicate samples were assigned replicate numbers 1 through 5. Each peptide identification is listed separately for each replicate number in which the peptide was observed.
(XLSX)

S2 Table Ontological grouping of α7-nAChR associated proteins independent of Ric-3 expression. Receptor protein complexes were eluted from α-bgtx-affinity beads complexes using carbachol, reduced, alkylated, precipitated and digested with trypsin in solution. Tryptic fragments were analyzed using a Q Exactive mass spectrometer. Ninety-seven proteins were identified as interacting with α7-nAChR only in the absence of Ric-3 expression by comparing α-bgtx isolated proteins from SH-EP1-hα7 samples SH-EP1-hα7-Ric-3 samples. Each condition was analyzed with five replicates. Data analysis was performed using ProteoIQ version 2.7 Protein inclusion criteria include 1% protein FDR, minimum peptide length of six amino acids ≥90% probability, identification in 2 or more of 5 replicates, and 0% probability in controls. FDRs were determined using the PROVALT algorithm and probabilities were determined with the ProteinProphet algorithm through ProteoIQ analysis. Only Top and Co-Top identifications were considered. Biological processes are listed as determined by DAVID analysis of Gene Ontology (GO) terms. Biological process GO terms for six proteins are not available.
(XLSX)

S3 Table Proteins identified in α-bgtx-affinity enriched samples from SH-EP1-hα7-Ric-3 and SH-EP1-hα7. 625 identified proteins meeting the established inclusion criteria were identified in both SH-EP1-hα7-Ric-3 and SH-EP1-hα7 samples. These proteins represent both possible α7-nAChR interactions regardless of Ric-3 expression as well as non-specific binding to α-bgtx-affinity beads. Further investigation is required to distinguish which identifications are non-specific. Data analysis was performed using ProteoIQ version 2.7 Protein inclusion criteria include 1% protein FDR, minimum peptide length of six amino acids ≥90% probability, identification in 2 or more of 5 replicates, and 0% probability in controls. FDRs were determined using the PROVALT algorithm and probabilities were determined with the ProteinProphet algorithm through ProteoIQ analysis. Only Top and Co-Top identifications were considered.
(XLSX)

S4 Table Cellular compartment GO terms associated with identified proteins. GO terms for cellular localization of identified SH-EP1-hα7-Ric-3 unique (A) and SH-EP1-hα7 unique (B) proteins. A total of 82% of the 39 (32 total) SH-EP1-hα7-Ric-3 unique, Ric-3-mediated proteins and 83% of the 97 (82 total) SH-EP1-hα7 unique proteins have GO terms that identifies the cellular compartment where the proteins have been reported to be localized. Proteins are identified by Uniprot accession numbers. The number of proteins associated with each compartment is listed as “Protein count” and the proportion of proteins classified into each compartment are listed as a percent of the total attributed proteins.
(XLSX)
