# Supplementary figures and images for: Resistance to Inhibitors of Cholinesterase 3 (Ric-3) Expression Promotes Selective Protein Associations with the Human α7-Nicotinic Acetylcholine Receptor Interactome
Source: PLoS One. 2015 Aug 10;10(8):e0134409. doi: 10.1371/journal.pone.0134409 (PMC4530945; doi:10.1371/journal.pone.0134409)

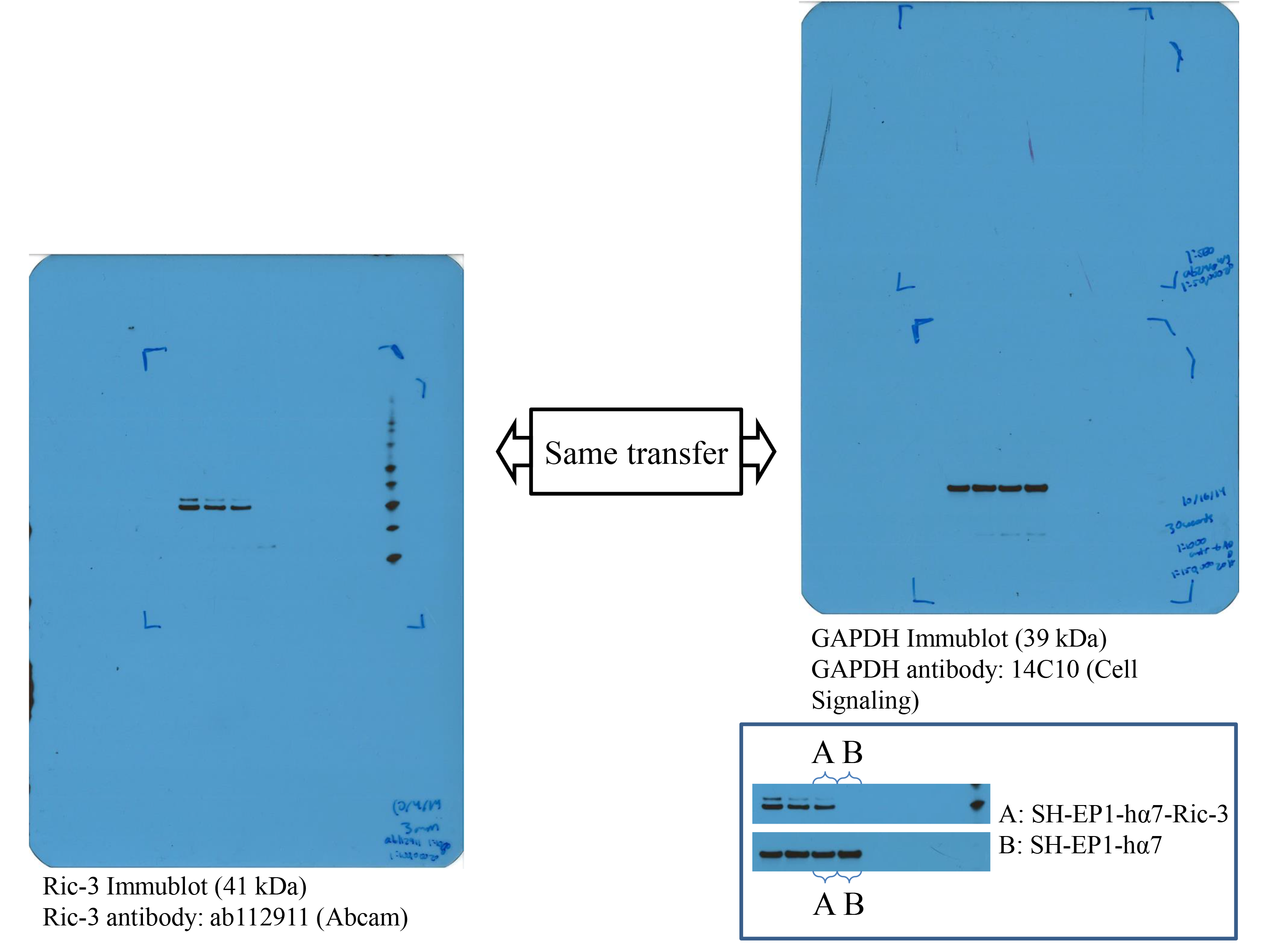

Supplement: S1 Fig — Immunoblots for investigation of Ric-3 and GAPDH immunoreactivity in SH-EP1-hα7-Ric-3 (A) and SH-EP1-hα7 (B) solubilized membrane extracts. Blots are initially probed for Ric-3 immunoreactivity, stripped, and subsequently probed again for GAPDH immunoreactivity to confirm consistent gel loading. (TIF) [file pone.0134409.s001.tif]
